# In Vivo Distribution and Therapeutic Efficacy of Radioiodine-Labeled pH-Low Insertion Peptide Variant 3 in a Mouse Model of Breast Cancer

**DOI:** 10.1155/2022/7456365

**Published:** 2022-07-04

**Authors:** Min Zhang, Yue Xi, Hong Chen, Wangxi Hai, Biao Li

**Affiliations:** Department of Nuclear Medicine, Ruijin Hospital, Shanghai Jiao Tong University School of Medicine, Shanghai, China

## Abstract

**Purpose:**

Extracellular acidity is a marker of highly aggressive breast cancer (BC). pH-low insertion peptides (pHLIPs) target the acidic tumor microenvironment. This study evaluates the distribution and therapeutic efficacy of radioiodine-labeled pHLIP variant 3 (Var3) in a mouse model of BC.

**Methods:**

The binding of fluorescein isothiocyanate (FITC)- or radioiodine-125 (^125^I) labeled Var3-pHLIP to MDA-MB-231, 4T1, and SK-BR-3 BC cell lines under different pH values was evaluated in vitro. The distribution of ^125^I-labeled Var3-pHLIP and wild-type- (WT-) pHLIP in tumor-bearing mice was analyzed in vivo using micro-SPECT/CT imaging. The therapeutic efficacy of radioiodine-131 (^131^I)-labeled Var3-pHLIP in MDA-MB-231 xenografts was evaluated by relative tumor volume measurement and immunohistochemical analysis.

**Results:**

The binding ability of FITC- or ^125^I-labeled Var3-pHLIP to tumor cells increased with the decrease in pH. The tumor-to-background ratio of ^125^I-Var3-pHLIP in BC xenografts showed the best imaging contrast at 24 h or 48 h postinjection. The uptake of ^125^I-Var3-pHLIP in MDA-MB-231 xenografts at 2 h postinjection was significantly higher than that of ^125^I-WT-pHLIP (3.76 ± 0.37 vs. 2.87 ± 0.60%ID/g, *p* = 0.046). The relative tumor volume in MDA-MB-231 xenografts was significantly lower in the ^131^I-Var3-pHLIP-treated group than in the groups treated with Var3-pHLIP (*p* = 0.027), ^131^I (*p* = 0.001), and saline (*p* < 0.001). The ^131^I-Var 3-pHLIP group presented a lower expression of Ki67 and a higher expression of caspase 3.

**Conclusion:**

Radioiodine-labeled Var3-pHLIP effectively targeted BC cells in an acidic environment and inhibited the growth of MDA-MB-231 xenografts by ionizing radiation.

## 1. Introduction

Breast cancer (BC) is the leading cause of cancer death among women [1]. Triple-negative BC (TNBC) is a highly aggressive BC subtype with a high rate of relapse and metastasis and a low survival rate [2]. Chemotherapy resistance is common in TNBC, and the efficacy of endocrine and antihuman epidermal growth factor receptor-2 (HER2) therapies is limited [3]. Therefore, new effective targeted therapies are needed.

Acidosis in the tumor microenvironment promoted by the Warburg effect is a hallmark of cancer. The extracellular pH in this environment is below 6.5 because of H^+^ production and excretion, whereas extracellular pH in healthy tissue under physiologic conditions is 7.2–7.4. In addition, the acidic microenvironment is not affected by clonal selection and thus is promising for diagnostic imaging and targeted therapy [4].

The 37-amino-acid pH-low insertion peptide (pHLIP) is water-soluble at pH ≥ 7.4 and has a low affinity for cell membranes. However, pHLIP is lipophilic under acidic conditions and forms an *α*-helix that crosses the cell membrane lipid bilayer [5]. Therefore, pHLIPs are used in fluorescence imaging [6–8], radionuclide imaging [9, 10], and drug delivery to the acidic tumor microenvironment [11, 12]. Variant 3- (Var3-) pHLIP has fast blood clearance and effectively targets cancer cells in primary tumors [9, 13, 14]. Labeling procedures using radionuclides such as technetium-99 m (^99m^Tc), iodine-125 (^125^I), and iodine-131 (^131^I) are simpler for in vivo imaging than labeling with positron-emitting nuclides. Furthermore, ^131^I can emit beta radiation for tumor therapy. ^99m^Tc-labeled wild-type- (WT-) pHLIP [15] and ^125^I-labeled pHLIP variant 7 [16] are promising for in vivo imaging of BC; however, radioiodine-labeled Var3-pHLIP has not been used in imaging and treatment of BC to date.

This study compared the in vivo distribution of ^125^I-WT-pHLIP and ^125^I-Var3-pHLIP in mice-bearing human BC xenografts and investigated the therapeutic efficacy of ^131^I-Var3-pHLIP in MDA-MB-231 xenografts.

## 2. Materials and Methods

### 2.1. Cell Culture

The human BC cell lines MDA-MB-231 (triple-negative BC), 4T1 (stage IV BC), and SK-BR-3 (HER2-positive BC) were purchased from The Cell Bank of the Chinese Academy of Sciences (Shanghai, China) and were cultured in DMEM (Gibco, NYC, USA) containing 4.5 g/L glucose, 10% fetal bovine serum (Gibco, NY, USA), and 1% penicillin-streptomycin (Sigma, St Louis, MO, USA). Cells were incubated at 37°C in a humidified incubator with 5% CO_2_.

### 2.2. Peptide Synthesis and Fluorescent Labeling

pHLIP was synthesized by GL Biochem Ltd. (Shanghai, China) through solid-phase chemical synthesis. The amino acid sequences of WT- and Var3-pHLIP are ACEQNPIYWARYADWLFTTPLLLLDLALLVDADEGTK and ACDDQNPWRAYLDLLFPTDTLLLDLLWK. The N-terminal of pHLIP was conjugated with fluorescein isothiocyanate (FITC), and fluorescence in vitro was measured at 495 nm excitation and 519 nm emission.

### 2.3. Peptide Labeling with ^125^I or ^131^I and In Vitro Stability

WT-pHLIP and Var3-pHLIP were labeled with ^125^I for in vivo imaging experiments, and Var3-pHLIP was labeled with ^131^I for therapeutic efficacy experiments. Radioactive iodine binds to tyrosine residues in pHLIPs. Peptides were dissolved in 200 *μ*L of 0.02 M PBS (pH 7.4) to reach a concentration of 1 mg/mL and mixed with 10 *μ*L of Na^125^I (74 MBq) or 20 *μ*L of Na^131^I (925 MBq). Chloramine-T (20 *μ*L, 5 mg/mL) was added to the test tubes and mixed for 70 s at room temperature. The reaction was stopped by adding 200 *μ*L of sodium metabisulfite (5 mg/mL). The peptides were eluted in ethanol through a C18 column equilibrated with 20 mL of 100% ethanol and 20 mL of distilled water. The radioactive purity of ^125^I-WT-pHLIP, ^125^I-Var3-pHLIP, and ^131^I-Var3-pHLIP was determined using thin-layer chromatography. Furthermore, the radiochemical purity of ^125^I-WT-pHLIP, ^125^I-Var3-pHLIP, and ^131^I-Var3-pHLIP was measured, and chemical stability was determined at 24 and 48 h at room temperature (25°C) in PBS and at 37°C in serum.

### 2.4. Fluorescence Imaging of FITC-Labeled Peptides In Vitro

DMEM medium and MES buffer were mixed at different volume ratios (100 : 0, 50 : 1, 25 : 2, 25 : 6, and 5 : 2) to simulate extracellular environments with a pH of 7.8, 7.4, 7.0, 6.6, and 6.2, respectively. MDA-MB-231, 4T1, and SK-BR-3 cells were seeded in 24-well plates at a density of 5 × 10^4^. After cell attachment, FITC-WT-pHLIP and FITC-Var3-pHLIP (50 *μ*g/mL) were added to the wells in triplicate. Cultures were incubated on a rotary shaker for 30 min. The culture medium was aspirated, and cells were washed three times with a solution of the respective pH without FITC-labeled peptides. Subsequently, 250 *μ*L of DAPI staining solution (100×) (Beyotime Biotechnology, Shanghai, China) diluted with DMEM medium at the respective pH was added to each well. The cells were incubated at 37°C in a humidified incubator with 5% CO_2_ for 10 min, washed three times with PBS at the respective pH, and imaged on a fluorescence microscope (×100).

### 2.5. In Vitro Cell Binding Assay

Cells were incubated with ^125^I-WT-pHLIP or ^125^I-Var3-pHLIP for 1 h at 37°C in the medium at different pH values ranging from 6.2 to 7.8 (6.2, 6.6, 7.0, 7.4, 7.8). At the same time, cells in the medium at pH 6.2 were further incubated with ^125^I-labeled peptide with an excess of unlabeled peptide (25 *μ*g/well). The supernatant was aspirated into a tube. The cells were then washed twice with PBS of the corresponding pH to remove unbound radioactivity. The wash solution was also added to the supernatant tube containing free radioactivity (*F*). The cells with bound radioactivity (*B*) were completely lysed with 300 *μ*l sodium hydroxide and aspirated into a cell tube. The radioactive counts of the cell and supernatant tube were measured by a *γ* counter. The cell binding fraction of the ^125^I-labeled peptides was calculated as *B*/(*B* + *F*) × 100%. All experiments were performed in triplicate.

### 2.6. Measurement of Cell Viability Using the Cell Counting Kit-8 (CCK-8) Assay

MDA-MB-231, 4T1, and SK-BR-3 cells were seeded in 96-well plates (5 × 10^3^ cells per well) and cultured for 12 hours. Cultures were divided into a treatment group (pHLIP [50 *μ*g/mL] in 100 *μ*L of culture medium at different pH values [7.8, 7.4, 7.0, 6.6, 6.2]) and control group (100 *μ*L of culture medium at the respective pH). The assays were performed in triplicate and repeated independently three times. The cells were incubated at 37°C in an incubator for 30 min, and the culture medium was aspirated. The CCK-8 solution was mixed with DMEM at a ratio of 1 : 9, and 100 *μ*L of the solution was added to each well. Absorbance was measured at 450 nm in a microplate reader. Absorbance in wells containing medium at pH 7.8 without pHLIP was defined as 100% cell viability, and the percentage of cell viability was calculated.

### 2.7. Animal Models

Five-week-old female athymic BALB/c nude mice (Shanghai Slaccas Experiment Animal Corp., Shanghai, China) were used in our study. Mouse xenograft models of MDA-MB-231, 4T1, and SK-BR-3 cells were produced by the subcutaneous injection of 5 × 10^6^ cells (suspended in 150 *μ*L PBS) into the right axilla of each mouse. The mice were used in subsequent experiments when tumor diameter reached 0.5-0.9 cm. The study protocol was approved by the institutional review board and by the Experimental Animal Center of Ruijin Hospital affiliated to the School of Medicine of Shanghai Jiao Tong University.

### 2.8. Micro-SPECT/CT Imaging of ^125^I-Labeled Peptides

Three days before micro-SPECT/CT imaging, 300 *μ*L of 1% Kl solution was administered to mice by oral gavage once a day, and 0.1% Kl was added into the drinking water to block thyroid gland activity. Mice-bearing MDA-MB-231, 4T1, or SK-BR-3 tumors were intravenously injected with 3.7 MBq of ^125^I-WT-pHLIP or ^125^I-Var3-pHLIP. Each group contained four mice. The mice were anesthetized by isoflurane inhalation, placed in the prone position, and scanned using a four-head SPECT/CT system (U-SPECT/CT, MI-Lab, Netherlands) and dedicated multipinhole apertures with a diameter of 1.5 mm at 1, 2, 4, 24, and 48 h after injection of ^125^I-labeled peptides. CT images were acquired after static SPECT imaging (10 min/frame). The images were reconstructed using U-SPECT REC software and analyzed using PMOD software version 3.9 (Switzerland). Regions of interest (ROIs) were drawn in the tumors and organs, including the brain, heart, liver, lungs, kidneys, intestine, and bladder, at various time points. The radioactivity per gram (ID%/g) in the ROIs was measured to obtain the dynamic tumor-to-background ratio (TBR, muscle tissue was used as the background) and the in vivo distribution and kinetics of ^125^I-WT-pHLIP and ^125^I-Var3-pHLIP in the xenograft models of BC.

### 2.9. Therapeutic Efficacy of ^131^I-Var3-pHLIP in MDA-MB-231 Xenografts

Twenty mice-bearing MDA-MB-231 human BC xenografts were divided into four groups of five animals. Group 1 was injected with ^131^I-Var3-pHLIP in the tail vein on days 0 and 3 (29.6 MBq each day). Groups 2, 3, and 4 were injected with the same volume of Var3-pHLIP, ^131^I (29.6 MBq), and saline solution, respectively. Tumor size was measured every 2 to 4 days postinjection (pi) for 26 days using a caliper. Tumor volumes (*V*, mm [3]) were calculated using the formula: *V* = (*a* × *b*^2^)/2, where *a* and *b* are tumor length and width, respectively. Relative tumor volumes were calculated as *V*/*V*_0_ (*V*_0_, tumor volume before treatment). The body weight of mice was also determined.

### 2.10. Immunohistochemical Analysis

The animals were sacrificed by cervical dislocation. The tumors were excised, fixed in paraformaldehyde, embedded in paraffin, and sectioned. The sections were mounted on glass slides, dewaxed in xylene, dehydrated through a graded ethanol series, and incubated in 3% hydrogen peroxide for 25 min. The samples were incubated with rabbit anti-human caspase 3 (1 : 100, Servicebio, Wuhan, China) and rabbit anti-human Ki67 (1 : 500, Servicebio, Wuhan, China) antibodies for 1 h at room temperature. Slices were washed with PBS thoroughly and incubated with HRP-labeled secondary antibody for 1 h at room temperature. Then, DAB kit was used for visualization and examined by light microscopy, and cells containing brown pigment were considered positively stained.

### 2.11. Statistical Analysis

Data were analyzed using the SPSS version 20.0 (SPSS Inc., Chicago, IL, USA) and GraphPad PRISM version 7.0 (GraphPad Software, La Jolla, CA, USA) and represented as means ± SD. Statistical significance was analyzed by one-way analysis of variance (ANOVA) with Bonferroni correction and was established at *p* < 0.05.

## 3. Results

### 3.1. Synthesis and Purification of Peptides

High-performance liquid chromatography analysis showed that the concentration of the main constituent of WT-pHLIP was 91.4% with a retention time of 14.9 min, whereas the concentration of the main component of Var 3-pHLIP was 95.7% with a retention time of 9.5 min. Mass spectrometry analysis showed that WT-pHLIP had a peak at m/z 1135.4 and a molecular weight of 4536.1 Da, and Var3-pHLIP had a peak at m/z 1240.3 and a molecular weight of 3724.2 Da.

### 3.2. Radiochemical Purity and In Vitro Stability

The radioactive purity of ^125^I-WT-pHLIP, ^125^I-Var3-pHLIP, and ^131^I-Var3-pHLIP was 99.6%, 98.9%, and 99.5%, respectively. At room temperature in PBS buffer, ^125^I-WT-pHLIP, ^125^I-Var3-pHLIP, and ^131^I-Var3-pHLIP had a stability of >95% at 24 h and >90% at 48 h. After 24 and 48 h at 37°C in serum, the stabilities were 86.8% and 86.6% for ^125^I-WT-pHLIP and 83.2% and 82.6% for ^125^I-Var3-pHLIP, respectively.

### 3.3. Fluorescence Imaging and Cell Viability at Different pH Values

**Figure 1:**
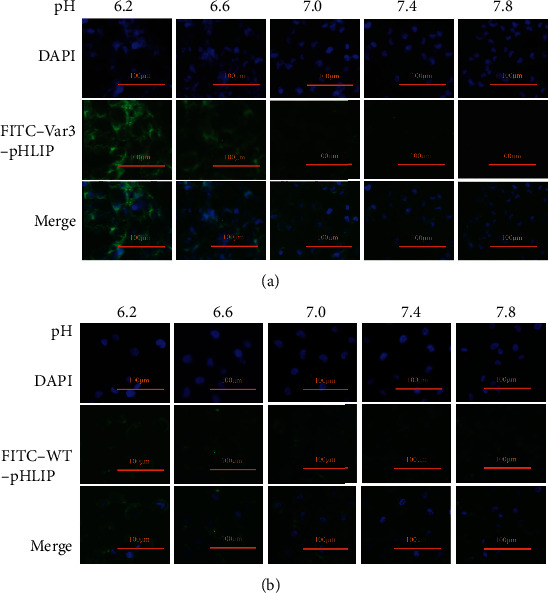
Fluorescence imaging and cell viability at different pH values. Fluorescence imaging (×100) demonstrated that the ability of (a) FITC-labeled variant 3 pH-low insertion peptide (var3-pHLIP) and (b) FITC-labeled wild-type (WT)-pHLIP to bind to MDA-MB-231 cell line increased with the decrease in pH (7.8, 7.4, 7.0, 6.6, and 6.2).

**Figure 2:**
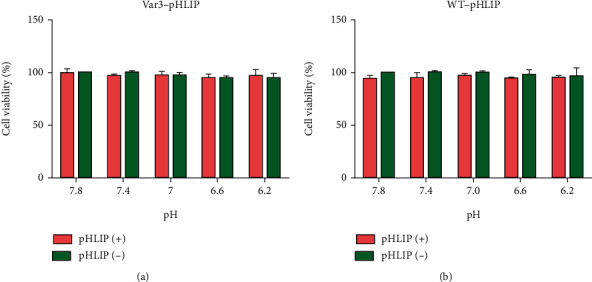
Cell Counting Kit-8 (CCK-8) assay showing cell viability. CCK8 assay showed no significant difference in cell viability in MDA-MB-231 cell line after treatment with (a) FITC-Var3-pHLIP and (b) FITC-WT-pHLIP under different pH conditions.

Fluorescence imaging showed that the in vitro distribution of FITC-Var3-pHLIP and FITC-WT-pHLIP in the cell membrane decreased from pH 6.2 to pH 7.8 in the cell lines MDA-MB-231 (Figures 1(a) and 1(b)), 4T1, and SK-BR-3 (Supplemental Figures 1(a)-1(b)). The CCK8 results showed no significant difference in cell viability between MDA-MB-231 (Figures 2(a) and 2(b)), 4T1, and SK-BR-3 (Supplemental Figure 2) treated with or without these two types of pHLIP at the same pH value. Furthermore, there was no significant difference in cell viability between pH treatments.

### 3.4. Binding Fractions of ^125^I-Labeled pHLIPs to Cells at Different pH Values

**Figure 3:**
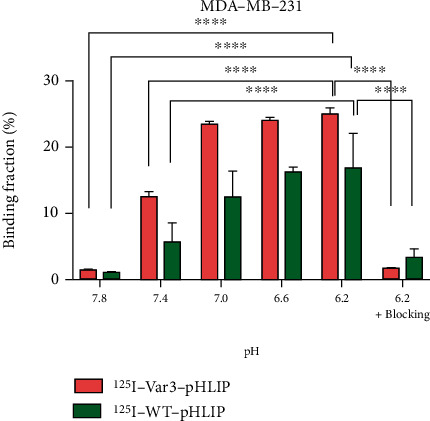
Binding fractions of ^125^I-labeled pHLIPs to MDA-MB-231 cells at different pH values. The binding fractions to MDA-MB-231 cells from pH 7.8 to 6.2 were 0.83 ± 0.21, 5.53 ± 2.88, 12.33 ± 3.97, 16.07 ± 0.81, and 16.70 ± 5.28 for ^125^I-WT-pHLIP and 1.30 ± 0.10, 12.3 ± 0.80, 23.40 ± 0.44, 23.88 ± 0.55, and 24.90 ± 0.96 for ^125^I-Var3-pHLIP, respectively. The cell binding fraction at pH 6.2 was significantly higher than those at pH 7.4 (*p* < 0.0001) and 7.8 (*p* < 0.0001). With an excess of unlabeled peptide, the binding fractions of ^125^I-WT-pHLIP or ^125^I-Var3-pHLIP to cells at pH 6.2, respectively, decrease to 3.11 ± 1.35% and 1.50 ± 0.11%. ^∗∗∗∗^*p* < 0.0001.

The binding fractions to MDA-MB-231 cells from pH 7.8 to 6.2 were 0.83 ± 0.21%, 5.53 ± 2.88%, 12.33 ± 3.97%, 16.07 ± 0.81%, and 16.70 ± 5.28% for ^125^I-WT-pHLIP and 1.30 ± 0.10%, 12.3 ± 0.80%, 23.40 ± 0.44%, 23.88 ± 0.55%, and 24.90 ± 0.96% for ^125^I-Var3-pHLIP, respectively, (Figure 3). The cell binding fraction at pH 6.2 was significantly higher than those at pH 7.4 (*p* < 0.0001) and 7.8 (*p* < 0.0001) suggesting pH-dependent binding of peptides to cells. Furthermore, with an excess of unlabeled peptide, the binding fractions of ^125^I-WT-pHLIP or ^125^I-Var3-pHLIP to MDA-MB-231 cells at pH 6.2, respectively, decrease to 3.11 ± 1.35% and 1.50 ± 0.11%, indicating binding specificity of ^125^I-labeled pHLIPs. Similar results were found for the trend of binding fraction of ^125^I-labeled pHLIPs to 4T1 or SK-BR-3 cells under pH gradient (Supplemental Figures 3(a)-3(b)).

### 3.5. In Vivo Imaging and Distribution of ^125^I-Labeled pHLIPs in Mice

**Figure 4:**
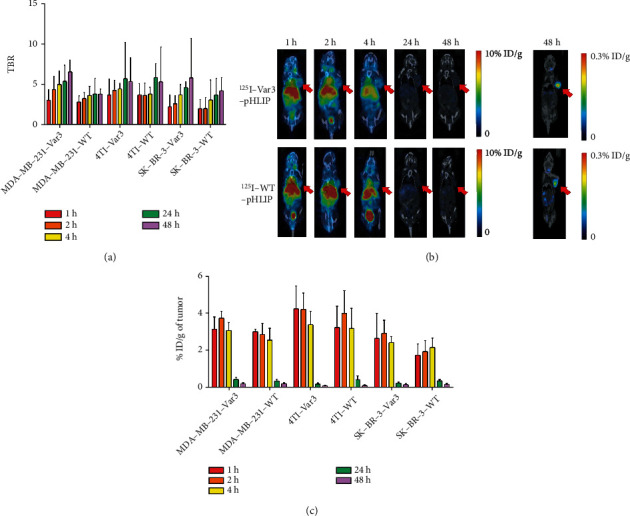
In vivo micro-SPECT/CT imaging and distribution of ^125^I-labeled pHLIPs in MDA-MB-231, SK-BR-3, and 4T1 breast cancer xenografts. (a) Tumor-to-background ratio (TBR) of ^125^I-labeled variant 3 (Var3)-pHLIP and ^125^I-labeled wild-type (WT)-pHLIP in xenografts peaked at 24-48 h after injection. The TBR of ^125^I-Var3-pHLIP was significantly higher than that of ^125^I-WT-pHLIP at 48 h in MDA-MB-231 xenografts (6.51 ± 1.45 vs. 3.76 ± 0.61, *p* = 0.013) but was not significantly different in 4T1 (5.32 ± 2.96 vs. 5.29 ± 4.29, *p* = 0.992) and SK-BR-3 (5.77 ± 4.92 vs. 4.18 ± 1.64, *p* = 0.562) xenografts. (b) In vivo micro-SPECT/CT imaging of ^125^I-Var3-pHLIP (upper) and ^125^I-WT-pHLIP (lower) in mice-bearing MDA-MB-231 tumors (red arrow). (c) The radioactive count of ^125^I-Var3-pHLIP and ^125^I-WT-pHLIP in xenografts peaked at 2 h after injection. Compared with ^125^I-WT-pHLIP, the uptake of ^125^I-Var3-pHLIP was significantly higher in MDA-MB-231 xenografts at 2 h pi (*p* = 0.046) but not in 4 T1 (*p* = 0.781) or SK-BR-3 xenografts (*p* = 0.077).

In vivo micro-SPECT/CT imaging at 1, 2, 4, 24, and 48 h after injection of ^125^I-pHLIP demonstrated that the TBR of ^125^I-Var3-pHLIP and ^125^I-WT-pHLIP was the highest at 24 or 48 h in the three types of xenografts, and imaging contrast was the highest at these two time points (Figures 4(a) and 4(b)). The TBR of ^125^I-Var3-pHLIP was significantly higher than that of ^125^I-WT-pHLIP at 48 h in MDA-MB-231 xenografts (6.51 ± 1.45 vs. 3.76 ± 0.61, *p* = 0.013) but was not significantly different in 4T1 (5.32 ± 2.96 vs. 5.29 ± 4.29, *p* = 0.992) and SK-BR-3 (5.77 ± 4.92 vs. 4.18 ± 1.64, *p* = 0.562) xenografts.

The distribution of ^125^I-Var3-pHLIP in tumors was the highest at 2 h pi (3.76 ± 0.37%ID/g in MDA-MB-231, 2.93 ± 0.70%ID/g in SK-BR-3, and 4.23 ± 0.88%ID/g in 4 T1) and decreased from 4 h to 48 h pi (3.09 ± 0.44, 0.47 ± 0.12, 0.22 ± 0.07%ID/g in MDA-MB-231; 2.45 ± 0.32, 0.26 ± 0.06, 0.18 ± 0.06%ID/g in SK-BR-3; and 3.40 ± 0.72, 0.21 ± 0.07, 0.11 ± 0.04%ID/g in 4T1) (Figure 4(c)). Compared with ^125^I-WT-pHLIP (2.87 ± 0.60%ID/g), the maximum uptake of ^125^I-Var3-pHLIP was significantly higher in MDA-MB-231 xenografts at 2 h pi (*p* = 0.046). However, there was no statistical uptake difference of these two pHLIPs at the same time in 4T1 (4.01 ± 1.23 vs. 4.23 ± 0.87, *p* = 0.781) or SK-BR-3 (1.96 ± 0.59 vs. 2.93 ± 0.71, *p* = 0.077) xenografts.

The radioactive count in major organs or tissues of MDA-MB-231 tumor-bearing nude mice (Tables 1 and 2) and other two BC mice models (Supplemental Table 1-4) decreased over time. The uptake of ^125^I-Var3-pHLIP and ^125^I-WT-pHLIP was high in the heart, liver, lungs, bladder, and kidneys.

### 3.6. Therapeutic Efficacy of ^131^I-pHLIP (Var3) In Vivo

**Figure 5:**
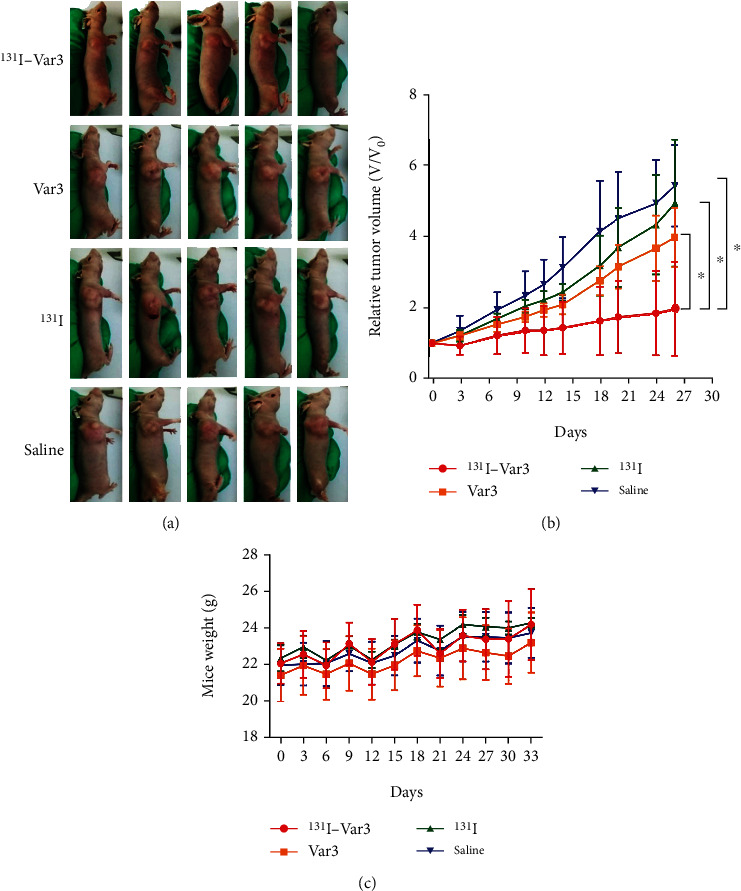
Therapeutic efficacy of ^131^I-labeled variant 3 pH-low insertion peptide (Var3-pHLIP) in mice-bearing MDA-MB-231 xenografts. (a) Images of MDA-MB-231 xenografts in the four study groups (*n* = 5 for each group) at the end of treatment. (b) The relative tumor volume in MDA-MB-231 xenografts was significantly lower in the ^131^I-Var3-pHLIP-treated group (2.06 ± 0.47) than in the groups treated with Var3-pHLIP (4.17 ± 0.51, *p* = 0.027), ^131^I (5.14 ± 1.51, *p* = 0.001), and saline (5.43 ± 1.15, *p* < 0.001) at the end of treatment. The asterisk indicates that *p* value is less than 0.05. (c) There was no significant difference in body weight among the groups. Data are means ± SD.

**Figure 6:**
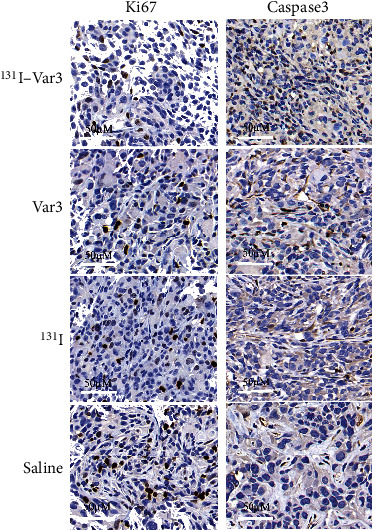
The immunohistochemical expression of Ki67 and caspase 3. The Ki67 expression was lower, and the caspase 3 expression was higher in the ^131^I-Var3-pHLIP-treated group than in the other three groups (magnification, ×365).

The relative tumor volume in MDA-MB-231 xenografts was significantly lower in the ^131^I-Var3-pHLIP-treated group (2.06 ± 0.47) than that in the groups treated with Var-pHLIP (4.17 ± 0.51, *p* = 0.027), ^131^I (5.14 ± 1.51, *p* = 0.001), and saline (5.43 ± 1.15, *p* < 0.001) at the end of treatment (Figures 5(a) and 5(b)). There was no significant difference in body weight among the groups (Figure 5(c)). Moreover, immunohistochemical staining showed that the Ki67 expression was lower, and the caspase 3 expression was higher in ^131^I-Var3-pHLIP-treated mice than in the other three groups (Figure 6), suggesting that ^131^I-Var3-pHLIP reduced tumor growth and increased apoptosis in MDA-MB-231 xenografts.

## 4. Discussion

The increased acidity in the extracellular environment due to the Warburg effect in highly aggressive cancers, including TNBC, where endocrine and molecular therapies are ineffective, is a new therapeutic target. pHLIPs sense the pH in the vicinity of the plasma membrane. The protonation of two aspartate residues in the spanning domain of pHLIP in an acidic environment allows the insertion of this peptide across the plasma membrane [12].

WT-pHLIP, Var3-pHLIP, and Var7-pHLIP are currently the most promising peptides of the pHLIP family. Positron emission tomography and fluorescence studies showed that, compared with WT-pHLIP, the distribution of Var3-pHLIP and Var7-pHLIP in the liver was lower probably because of their lower hydrophobicity at physiological pH. Var7-pHLIP is shorter and less hydrophobic than Var3-pHLIP, increasing clearance from the blood and liver but potentially decreasing its uptake by tumors [9, 14]. Therefore, Var3-pHLIP was selected for this study. A previous study showed that the uptake of [17]F and ^64^Cu-labeled Var3-pHLIP in 4 T1 xenografts was 10.6 ± 2.3%ID/g at 4 h pi and 19.6 ± 2.3%ID/g at 24 h pi, respectively, and the TBR was 6.9 ± 1.9 and 16.0 ± 3.0, respectively, showing the highest tumoral uptake and tumor-to-background contrast among the three pHLIPs [9]. Our results showed that the tumor uptake and TBR of ^125^I-Var3-pHLIP were higher than those of ^125^I-WT-pHLIP; however, the significance differed among the three cell lines. The maximum uptake of ^125^I-Var3-pHLIP was earlier (2 h vs. 24-48 h) and lower than that of ^64^Cu-Var3-pHLIP. Similarly, the maximum uptake of ^125^I-Var7-pHLIP was at 1 h pi [16], and the near-infrared fluorescence of this pHLIP was the highest at 2-4 h pi. The discrepancy in maximum uptake may be due to differences in the labeling methods. The labeling of pHLIPs with positron-emitting nuclides requires the addition of a chelating agent, which may reduce hydrophobicity and increase peptide aggregation around tumor cell membranes. The replacement of the hydrogen atom on the phenyl ring of the tyrosine residue with ^125^I or ^131^I during the iodination of Var3-pHLIP using the chloramine-T method has little effect on peptide hydrophobicity.

The ability of pHLIP to target the acidic microenvironment of tumors allows its use as drug carriers. Studies have covalently bound therapeutic drugs that cannot cross the plasma membrane (phallacidin [18], amanitin [17], protease-activated receptor 1-activating peptide [19], antimiR-155 [11]) to the hydroxyl terminus of pHLIP, allowing drug release to tumors. ^131^I labeling is simpler than conjugating the above drugs to pHLIP. The present study showed that ^131^I-Var3-pHLIP inhibited tumor growth in MDA-MB-231 xenografts through ionizing radiation damage. However, treatment had some limitations. First, ^131^I-Var3-pHLIP was widely distributed in blood-rich organs such as the liver, heart, and lungs, increasing radiation damage to these normal organs. Second, ^131^I-Var3-pHLIP was cleared quickly from tumors, and its distribution in tumor tissue at 24 h pi was 12.5% of that at 2 h pi; thus, repeated injections are necessary to achieve the radiation dose required for effective treatment. Therefore, developing pHLIP variants with higher retention in tumor and faster blood clearance is necessary to improve the therapeutic efficacy and safety of ^131^I-labeled pHLIPs. Third, previous studies have shown that local inflammation was closely associated with increased acidity of extracellular microenvironment in the involved organs and tissues, which makes pHLIPs accumulate in the inflammatory diseases such as arthritis [7], pneumonia [20], and active vulnerable plaques [21]. Thus, inflammatory disease may increase the interpretation complexity of pHLIP for cancer imaging and tumor selectivity of pHLIP as a therapeutic vehicle.

## 5. Conclusions

Radioiodine-labeled Var3-pHLIP effectively targeted breast cancer cells in an acidic environment and inhibited the growth of MDA-MB-231 xenografts by ionizing radiation.

## Figures and Tables

**Table 1:** Distribution of ^125^I-Var3-pHLIP in MDA-MB-231 tumor-bearing nude mice (%ID/g, *n* = 4).

Tissues or Organs		Time pi				
		1 h	2 h	4 h	24 h	48 h
Tumor		3.16 ± 0.67	3.76 ± 0.37	3.09 ± 0.44	0.47 ± 0.12	0.22 ± 0.07
Brain		1.02 ± 0.08	1.13 ± 0.12	0.75 ± 0.27	0.13 ± 0.07	0.09 ± 0.05
Heart		9.47 ± 1.54	8.29 ± 0.52	5.35 ± 0.93	0.39 ± 0.13	0.14 ± 0.05
Liver		10.34 ± 1.33	10.21 ± 0.66	8.11 ± 1.63	1.00 ± 0.54	0.40 ± 0.22
Lung		9.64 ± 0.80	9.10 ± 0.61	6.60 ± 0.88	0.49 ± 0.22	0.18 ± 0.05
Kidney		5.66 ± 2.26	5.61 ± 0.71	3.80 ± 0.89	0.41 ± 0.04	0.25 ± 0.13
Intestine		2.59 ± 0.50	3.18 ± 0.35	2.12 ± 0.33	0.20 ± 0.09	0.10 ± 0.06
Bladder		20.64 ± 5.71	24.75 ± 3.51	31.64 ± 15.95	2.14 ± 0.88	0.45 ± 0.14
Muscle		1.12 ± 0.28	0.96 ± 0.32	0.67 ± 0.21	0.09 ± 0.03	0.04 ± 0.01

**Table 2:** Distribution of ^125^I-WT-pHLIP in MDA-MB-231 tumor-bearing nude mice (%ID/g, *n* = 4).

Tissues or organs	Time pi				
	1 h	2 h	4 h	24 h	48 h
Tumor	3.03 ± 0.14	2.87 ± 0.60	2.57 ± 0.64	0.38 ± 0.10	0.24 ± 0.06
Brain	1.11 ± 0.21	1.03 ± 0.21	0.60 ± 0.13	0.17 ± 0.09	0.16 ± 0.10
Heart	10.74 ± 0.93	8.78 ± 1.15	5.76 ± 0.45	0.37 ± 0.13	0.10 ± 0.02
Liver	15.80 ± 2.29	14.95 ± 3.73	10.61 ± 2.69	1.21 ± 0.39	0.38 ± 0.09
Lung	13.26 ± 1.80	14.12 ± 2.38	7.93 ± 1.07	0.69 ± 0.17	0.27 ± 0.10
Kidney	6.96 ± 1.03	6.00 ± 0.8	4.13 ± 0.53	0.38 ± 0.31	0.16 ± 0.13
Intestine	3.17 ± 0.97	2.50 ± 0.16	2.92 ± 0.53	0.20 ± 0.12	0.12 ± 0.09
Bladder	20.44 ± 15.07	28.70 ± 6.66	18.76 ± 22.03	1.22 ± 0.99	0.27 ± 0.20
Muscle	1.14 ± 0.31	0.92 ± 0.26	0.74 ± 0.13	0.12 ± 0.04	0.07 ± 0.02

## Data Availability

The datasets generated and analyzed during the current study are available from the corresponding author on reasonable request.

## Supplementary Materials

Supplementary materials included fluorescence imaging showing the in vitro distribution of FITC-Var3-pHLIP and FITC-WT-pHLIP in the cell membrane from pH 6.2 to pH 7.8 in the cell lines of 4T1 and SK-BR-3 (Supplemental Figures 1(a)-1(b)), CCK8 results showing cell viability of 4T1 and SK-BR-3 (Supplemental Figure 2) treated with or without these two types of pHLIP at the same pH value, binding fractions of ^125^I-labeled pHLIPs to 4T1 and SK-BR-3 cells at different pH values (Supplemental Figures 3a-3b), and the radioactive count in major organs or tissues of 4T1 and SK-BR-3 tumor-bearing mice models (Supplemental Table 1-4).
