# Supplementary material for: In Vivo Distribution and Therapeutic Efficacy of Radioiodine-Labeled pH-Low Insertion Peptide Variant 3 in a Mouse Model of Breast Cancer
Source: Mol Imaging. 2022 Jul 4;2022:7456365. doi: 10.1155/2022/7456365 (PMC9281440; doi:10.1155/2022/7456365)
Supplement: Supplementary materials — included fluorescence imaging showing the in vitro distribution of FITC-Var3-pHLIP and FITC-WT-pHLIP in the cell membrane from pH 6.2 to pH 7.8 in the cell lines of 4T1 and SK-BR-3 (Supplemental Figures 1(a)-1(b)), CCK8 results showing cell viability of 4T1 and SK-BR-3 (Supplemental Figure 2) treated with or without these two types of pHLIP at the same pH value, binding fractions of 125I-labeled pHLIPs to 4T1 and SK-BR-3 cells at different pH values (Supplemental Figures 3a-3b), and the radioactive count in major organs or tissues of 4T1 and SK-BR-3 tumor-bearing mice models (Supplemental Table 1-4). [file 7456365.f1.zip › Supplemental tables.docx]

Supplemental Table1

Distribution of ^125^I-Var3-pHLIP in 4T1 tumor-bearing nude mice (%ID/g, n=4)

| Tissues or Organs | Time pi | | | | |
| --- | --- | --- | --- | --- | --- |
|  | 1h | 2h | 4h | 24h | 48h |
| Tumor | 4.26±1.24 | 4.23±0.88 | 3.40±0.73 | 0.21±0.07 | 0.12±0.04 |
| Brain | 1.44±0.24 | 1.32±0.49 | 1.04±0.47 | 0.16±0.10 | 0.13±0.09 |
| Heart | 14.41±2.44 | 10.96±2.39 | 8.21±2.03 | 0.36±0.16 | 0.17±0.05 |
| Liver | 14.89±3.55 | 13.16±2.28 | 12.56±3.61 | 0.60±0.16 | 0.23±0.04 |
| Lung | 13.85±3.09 | 11.27±3.09 | 9.40±2.45 | 0.48±0.15 | 0.19±0.10 |
| Kidney | 7.98±3.12 | 7.97±2.23 | 4.99±2.04 | 0.39±0.3 | 0.20±0.13 |
| Intestine | 3.65±0.64 | 3.63±1.19 | 3.23±0.70 | 0.28±0.15 | 0.14±0.11 |
| Bladder | 33.66±21.12 | 24.95±10.79 | 42.67±17.58 | 2.83±1.65 | 0.21±0.13 |
| Muscle | 1.41±0.61 | 1.03±0.19 | 0.80±0.26 | 0.06±0.04 | 0.03±0.03 |

Supplemental Table2

Distribution of ^125^I-WT-pHLIP in 4T1 tumor-bearing nude mice (%ID/g, n=4)

| Tissues or Organs | Time pi | | | | |
| --- | --- | --- | --- | --- | --- |
|  | 1h | 2h | 4h | 24h | 48h |
| Tumor | 3.25±1.15 | 4.01±1.23 | 3.21±1.07 | 0.45±0.19 | 0.13±0.04 |
| Brain | 1.26±0.60 | 1.02±0.42 | 0.75±0.39 | 0.18±0.14 | 0.14±0.09 |
| Heart | 12.29±5.41 | 9.33±4.09 | 6.59±3.11 | 0.46±0.11 | 0.24±0.24 |
| Liver | 14.58±2.98 | 12.66±3.04 | 13.62±5.08 | 0.98±0.3 | 0.28±0.08 |
| Lung | 13.05±3.22 | 11.81±3.09 | 9.75±2.01 | 0.73±0.09 | 0.21±0.11 |
| Kidney | 7.30±2.63 | 5.92±2.41 | 4.46±1.60 | 0.27±0.09 | 0.12±0.05 |
| Intestine | 3.56±1.33 | 3.26±0.98 | 2.80±1.56 | 0.22±0.10 | 0.13±0.10 |
| Bladder | 23.04±5.94 | 40.57±6.76 | 40.32±26.22 | 3.03±2.04 | 0.64±0.96 |
| Muscle | 0.93±0.35 | 1.17±0.33 | 0.84±0.20 | 0.09±0.05 | 0.05±0.04 |

Supplemental Table3

Distribution of ^125^I-Var3-pHLIP in SK-BR-3 tumor-bearing nude mice (%ID/g, n=4)

| Tissues or Organs | Time pi | | | | |
| --- | --- | --- | --- | --- | --- |
|  | 1h | 2h | 4h | 24h | 48h |
| Tumor | 2.68±1.34 | 2.94±0.71 | 2.45±0.32 | 0.26±0.06 | 0.18±0.06 |
| Brain | 1.02±0.07 | 0.95±0.07 | 0.68±0.20 | 0.09±0.02 | 0.09±0.01 |
| Heart | 10.27±0.54 | 8.89±0.18 | 6.02±1.35 | 0.38±0.08 | 0.10±0.02 |
| Liver | 11.38±0.87 | 10.36±1.60 | 11.40±2.75 | 0.81±0.20 | 0.23±0.02 |
| Lung | 9.81±0.82 | 9.31±1.03 | 6.93±1.24 | 0.42±0.12 | 0.14±0.05 |
| Kidney | 6.67±1.07 | 4.24±0.97 | 3.56±0.65 | 0.42±0.11 | 0.18±0.10 |
| Intestine | 3.05±0.68 | 3.01±0.26 | 2.38±0.68 | 0.31±0.14 | 0.07±0.04 |
| Bladder | 18.16±4.14 | 18.67±15.62 | 23.36±6.56 | 2.44±2.14 | 0.21±0.21 |
| Muscle | 1.33±0.30 | 1.21±0.26 | 0.70±0.12 | 0.06±0.02 | 0.04±0.02 |

Supplemental Table4

Distribution of ^125^I-WT-pHLIP in SK-BR-3 tumor-bearing nude mice (%ID/g, n=4)

| Tissues or Organs | Time pi | | | | |
| --- | --- | --- | --- | --- | --- |
|  | 1h | 2h | 4h | 24h | 48h |
| Tumor | 1.76±0.60 | 1.96±0.59 | 2.18±0.50 | 0.38±0.08 | 0.19±0.07 |
| Brain | 0.93±0.20 | 0.95±0.10 | 0.72±0.05 | 0.14±0.04 | 0.06±0.04 |
| Heart | 10.71±1.38 | 6.96±1.73 | 5.01±0.51 | 0.44±0.09 | 0.11±0.06 |
| Liver | 14.53±2.81 | 12.63±0.85 | 10.77±1.89 | 1.19±0.15 | 0.32±0.05 |
| Lung | 12.94±1.57 | 11.08±0.99 | 8.34±1.23 | 0.67±0.22 | 0.21±0.05 |
| Kidney | 6.65±1.68 | 4.70±0.77 | 4.46±1.19 | 0.34±0.11 | 0.09±0.03 |
| Intestine | 2.63±0.41 | 2.42±0.16 | 2.15±0.32 | 0.22±0.07 | 0.09±0.04 |
| Bladder | 24.73±11.72 | 22.88±7.71 | 29.43±28.79 | 1.87±1.16 | 0.25±0.04 |
| Muscle | 0.97±0.27 | 1.14±0.36 | 0.95±0.40 | 0.14±0.10 | 0.06±0.05 |
