# Supplementary figures and images for: In Vivo Distribution and Therapeutic Efficacy of Radioiodine-Labeled pH-Low Insertion Peptide Variant 3 in a Mouse Model of Breast Cancer
Source: Mol Imaging. 2022 Jul 4;2022:7456365. doi: 10.1155/2022/7456365 (PMC9281440; doi:10.1155/2022/7456365)

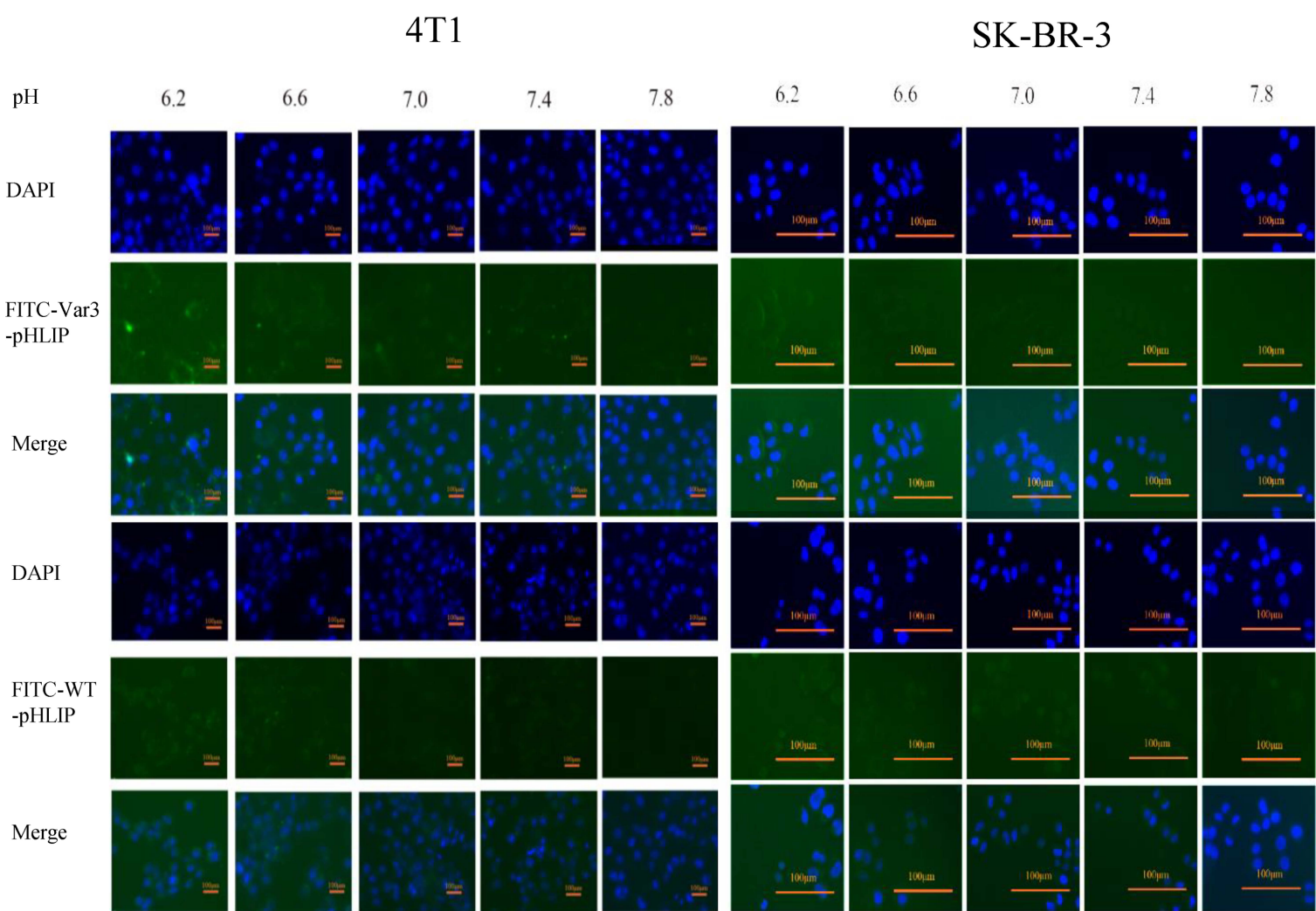

Supplement: Supplementary materials — included fluorescence imaging showing the in vitro distribution of FITC-Var3-pHLIP and FITC-WT-pHLIP in the cell membrane from pH 6.2 to pH 7.8 in the cell lines of 4T1 and SK-BR-3 (Supplemental Figures 1(a)-1(b)), CCK8 results showing cell viability of 4T1 and SK-BR-3 (Supplemental Figure 2) treated with or without these two types of pHLIP at the same pH value, binding fractions of 125I-labeled pHLIPs to 4T1 and SK-BR-3 cells at different pH values (Supplemental Figures 3a-3b), and the radioactive count in major organs or tissues of 4T1 and SK-BR-3 tumor-bearing mice models (Supplemental Table 1-4). [file 7456365.f1.zip › Supplemental figure 1.pdf]

4T1

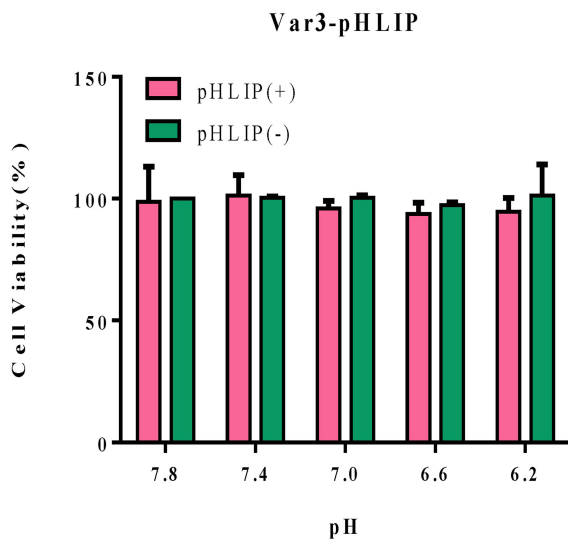

SK-BR-3

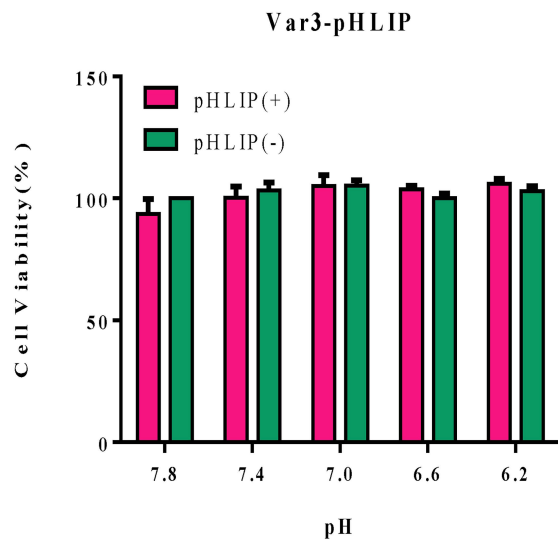

**WT-pHLIP**

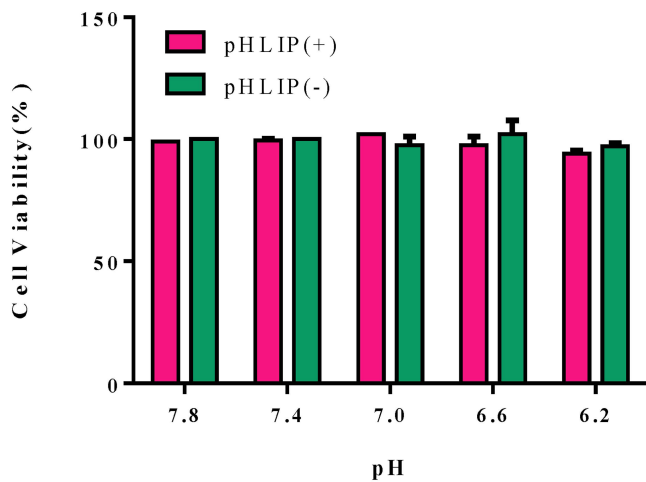

**WT-pHLIP**

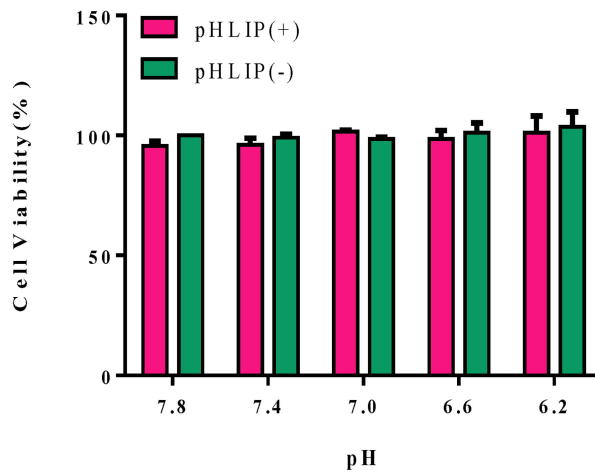

Supplement: Supplementary materials — included fluorescence imaging showing the in vitro distribution of FITC-Var3-pHLIP and FITC-WT-pHLIP in the cell membrane from pH 6.2 to pH 7.8 in the cell lines of 4T1 and SK-BR-3 (Supplemental Figures 1(a)-1(b)), CCK8 results showing cell viability of 4T1 and SK-BR-3 (Supplemental Figure 2) treated with or without these two types of pHLIP at the same pH value, binding fractions of 125I-labeled pHLIPs to 4T1 and SK-BR-3 cells at different pH values (Supplemental Figures 3a-3b), and the radioactive count in major organs or tissues of 4T1 and SK-BR-3 tumor-bearing mice models (Supplemental Table 1-4). [file 7456365.f1.zip › Supplemental figure 2.pdf]

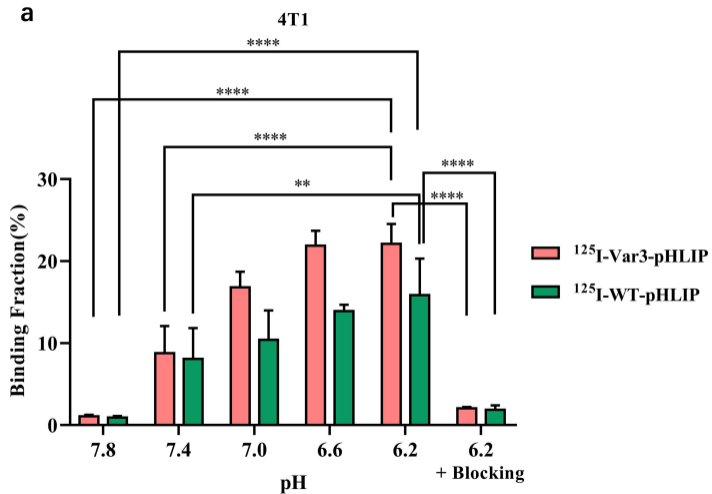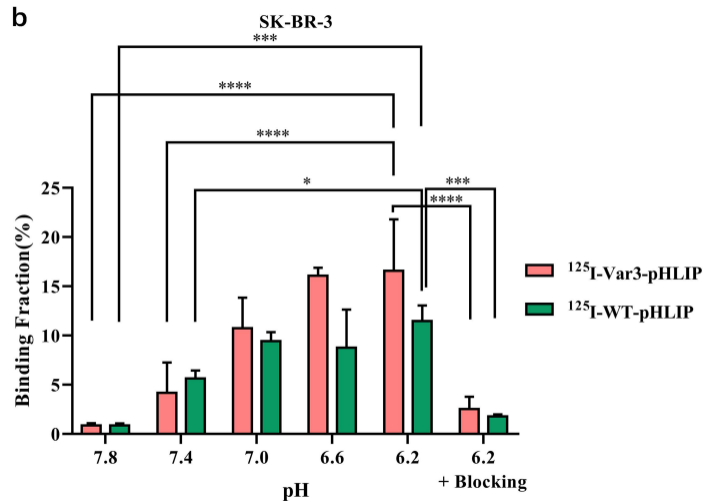

Supplement: Supplementary materials — included fluorescence imaging showing the in vitro distribution of FITC-Var3-pHLIP and FITC-WT-pHLIP in the cell membrane from pH 6.2 to pH 7.8 in the cell lines of 4T1 and SK-BR-3 (Supplemental Figures 1(a)-1(b)), CCK8 results showing cell viability of 4T1 and SK-BR-3 (Supplemental Figure 2) treated with or without these two types of pHLIP at the same pH value, binding fractions of 125I-labeled pHLIPs to 4T1 and SK-BR-3 cells at different pH values (Supplemental Figures 3a-3b), and the radioactive count in major organs or tissues of 4T1 and SK-BR-3 tumor-bearing mice models (Supplemental Table 1-4). [file 7456365.f1.zip › Supplemental figure 3.pdf]
